# A pathogenic and recombinant infectious bronchitis virus variant (CK/CH/GX/202109) with multiorgan tropism

**DOI:** 10.1186/s13567-023-01182-w

**Published:** 2023-07-03

**Authors:** Chenyan Wang, Bo Hou

**Affiliations:** Institute of Animal Husbandry and Veterinary Medicine, Fujian Academy of Agricultural Sciences/Fujian Animal Disease Control Technology Development Center, Fuzhou, 350013 Fujian Province China

**Keywords:** Infectious bronchitis virus, proventriculus, recombination, tissue tropism

## Abstract

Despite vaccine use, novel strains and variants of infectious bronchitis virus (IBV) have emerged continuously, leading to economic losses to the poultry industry worldwide. This study aimed to characterize the IBV isolate CK/CH/GX/202109 from three yellow broilers in Guangxi, China. Recombination was shown to have occurred in regions of the *1ab* gene. Compared to the whole genome of ck/CH/LGX/130530, which is genotypically related to tl/CH/LDT3-03, the 202109 strain had 21 mutations. The pathological assessment showed that this variant caused 30% and 40% mortality in 1-day-old chicks infected with oral and ocular inoculum, respectively. Nephritis, enlarged proventriculus, inflammation of the gizzard, and atrophy of the bursa of Fabricius were also observed at both 7 and 14 days post-infection (dpi). Viral loads in the trachea, proventriculus, gizzard, kidney, bursa, and cloacal swabs were higher at 7 dpi than at 14 dpi. Clinicopathological and immunohistochemical analyses revealed that this virus exhibited multiple organ tropisms capable of infecting the trachea, proventriculus, gizzard, kidney, bursa, ileum, jejunum, and rectum. Almost none of the 1-day-old infected chicks seroconverted until 14 dpi. While the virus was found in the ileum, jejunum, and rectum in the 28-day-old ocular group, the majority of 28-day-old infected chickens seroconverted at 10 dpi. These study findings demonstrate that recombination events and mutations during the evolution of IBV may greatly alter tissue tropism and emphasize the need for the continued surveillance of novel strains and variants in order to control this infection.

## Introduction

Infectious bronchitis virus (IBV) is an enveloped, single-stranded RNA virus belonging to the *Gammacoronavirus* genus and is a major cause of economic losses in the poultry industry worldwide. IBV infects chickens of all ages [1], and the clinical signs include respiratory distress [2], nephritis [3], proventriculitis [4], enteritis [5], and “false layer” syndrome [6]. Decreased egg production, reduced growth rate, and high morbidity are also associated with IBV infection [7, 8]. The IBV genome is 27.6 kb in length and encodes at least 10 open reading frames (ORFs), arranged in the 5′ to 3′ direction as UTR-1a/1ab-S-3a-3b-E-M-5a-5b-N-3′-UTR-poly(A). Four genes encode structural proteins, including the spike protein (S), the envelope (E), the matrix (M), and the nucleocapsid (N). At least two-thirds of the genome is occupied by the two genes, ORF1a and ORF1b, which are expressed as polyproteins 1a (pp1a) and 1ab (pp1ab), respectively. Proteolytic cleavage of the polyproteins yields 15 functional non-structural proteins [9]. The S protein is a transmembrane protein and is typically cleaved into two distinct polypeptides named S1 and S2. The S1 protein determines receptor binding [10] and is an important immunogenic component [11]. Phylogenetic analysis of the *S1* gene is used to define the IBV genotypes GI–GVII [12]. Conversely, the S2 subunit is potentially a determinant of cellular tropism [13]. The IBV genome also encodes accessory genes 3 and 5, which have been demonstrated to play an indispensable role in virus replication in cell culture [14] and pathogenicity [15, 16].

The generation of genetic diversity and the subsequent selection processes are the most important drivers of IBV evolution. Despite the 3′ to 5′ exoribonuclease proofreading function of non-structural protein 14 (nsp14) in coronavirus [17], the viral RNA mutation rate ranges from 10^–6^ to 10^–4^ substitutions per round of copying [18]. Mutations and high-frequency recombination are common phenomena during the IBV genome replication process and have given rise to different strains of IBV, therefore increasing the potential for new strains to arise from vaccinated flocks [2, 19]. Thus, novel or variant IBV strains have been frequently isolated following immune failure [20, 21]. The selection pressures introduced by the use of vaccines, in addition to those exerted by the infected host microenvironment and physical and biosecurity-associated factors, have caused IBV to evolve rapidly [22].

The virus is shed from epithelial cells of the trachea, kidney, oviduct, intestine, and bursa of Fabricius [23]. IBV GI-19 viruses and tl/CH/LDT3/03 type strains are responsible for respiratory signs and nephritis [24], while GVI strains are responsible for respiratory signs [25]. However, differences and changes in tissue tropism have been identified in different strains of the same genotype [4, 26–28]. The reasons for differences in tissue tropism, multiorgan tropism, and pathogenicity between IBV strains remain unclear. In this study, gene analysis, recombination analysis, phylogenetic analysis, and pathogenicity tests of one IBV variant were performed to provide insight into the altered characteristics of IBV strains of the same genotype.

## Materials and methods

### Virus and electron microscopy observation

Forty proventriculus and gizzard samples were collected from 28-day-old, three-yellow broilers vaccinated with the H120 and 4/91 strains in Guangxi Province, China, from March to May 2020. The broilers exhibited depression and decreased feed intake, slow growth, and developed proventriculitis. Proventriculus and gizzard harvesting was conducted as previously described, and the virus was isolated from the tissue samples by grinding with liquid nitrogen [29] prior to filtration through a 0.45 μm filter membrane. Three blind passages were conducted using 10-day-old specific-pathogen-free (SPF) embryos, as reported previously [25]. Real-time polymerase chain reaction (RT‒PCR) or PCR analysis of allantoic fluid was performed to detect Newcastle disease virus (NDV), infectious laryngotracheitis virus (ILTV), avian leucosis virus (ALV), infectious bursal disease virus (IBDV), reovirus and adenovirus. The quantitative RT‒PCR (qRT‒PCR) assay for IBV measurement was performed as described previously [25]. Three limiting dilution passages were conducted, as reported previously [25]. IBV-positive allantoic fluid was harvested and designated as 202109 p0. The 50% embryo infectious dose (EID_50_) was calculated using the Reed-Muench method. Typical virus morphologic characteristics were observed using a Hitachi HT-7700 transmission electron microscope (Tokyo, Japan) at 80 kV. Sample preparation and examination methods were reported in a previous study [25].

All 1-day-old and 28-day-old SPF chickens and SPF embryonated eggs were purchased from Beijing Boehringer Ingelheim Vital Biotechnology Co., Ltd. (Beijing, China).

### RNA extraction, sequencing, and recombinant analysis

The RNA extraction method and RT‒PCR assay have been described previously [25]. The PCR mix contained 12.5 μL of GoTaq Green Master Mix (Promega, Madison, WI, USA), 1 μL of each full-length *S1* primer as described previously [21] (10 μM; S1Oligo5′: 5′- TGA AAA CTG AAC AAA AGA C -3′, IBV-275: 5′- GTA TGT ACT CAT CTG TAA C -3′), 2 μL of template cDNA, and 8.5 μL of ultrapure water. The thermocycling conditions were as follows: 95 ℃ for 5 min; 35 cycles of 95 ℃ for 30 s, 50 ℃ for 60 s, and 68 ℃ for 120 s; and a final extension step at 68 ℃ for 10 min. The PCR product was approximately 1900 bp in length. DNA fragments were purified and submitted to Fuzhou Biosune Inc. (Fuzhou, China) for sequencing. The qRT‒PCR assay was performed as previously described (25), and IBV-specific primers (IBV 5′GU391 and IBV 5′GL533) and probes (IBV 5′G) were used [30].

Phylogenetic analysis of the 202109 strain was performed based on the *S1* gene using 61 reference strains in MEGA version 7.0. Viral RNA of the whole genome and cDNA synthesis were performed as previously described [25]. Whole genome sequencing and recombination analyses were conducted as described previously [25]. Pairwise comparisons of the whole genomes of the CK/CH/GX/202109 and ck/CH/LGX/130530 strains were performed.

### Experimental tests

All animal experiments were approved by the Institute of Animal Husbandry and Veterinary Medicine of the Fujian Academy of Agricultural Sciences and were performed in accordance with animal ethics guidelines and approved protocols. All husbandry procedures were conducted in compliance with the Animal Welfare Act and the Guide for the Care and Use of Laboratory Animals (permission No. 2022-006). Feed and water were provided ad libitum. Sixty 1-day-old SPF chicks and 60 28-day-old SPF chickens were numbered, weighed, and randomly divided into three groups of 20 birds in the control, oral and ocular groups. The birds in the oral or ocular groups were challenged with 10^6^ EID_50_ of the isolate, and the control groups were injected with sterile allantoic fluid. Each group of 1-day-old and 28-day-old chickens received ocular inoculations (50 μL for the 1-day-olds and 100 μL for the 28-day-olds). In addition, each group of 1-day-old and 28-day-old chickens received oral inoculations (0.5 mL for the 1-day-olds and 1 mL for the 28-day-olds). The clinical symptoms of all chickens were evaluated twice daily after inoculation. The 1-day-old and 28-day-old birds were observed on days 14 and 28, respectively.

Blood was collected from the live birds by wing venipuncture and tested for IBV antibodies at 3, 5, 7, 10, and 14 dpi in the 1-day-old groups and 3, 5, 7, 10, 14, 21, and 28 dpi in the 28-day-old groups. Cloacal swabs were taken at 7 and 14 dpi in the 1-day-old groups (live chicks) and at 7, 14, 21, and 28 dpi in the 28-day-old groups to measure IBV by qRT‒PCR. Finally, all live chickens from each group were weighed, bled, and euthanized following injection with tiletamine hydrochloride and zolazepam hydrochloride (Virbac, Carros, France).

The proventriculus, gizzard, and bursa of Fabricius of each bird were weighed. Portions of these organs, in addition to portions of the kidney, duodenum, jejunum, caecum, ileum, and the upper half of the trachea, were harvested and fixed immediately in 10% neutral buffered formalin. The remaining portions of these organs, except for the intestines and tissues from dead birds, were stored at −70 °C for later viral load determination by qRT‒PCR. The relative organ weights were calculated using the following formula: relative organ weight = [organ weight (g)/body weight (g)] × 100 [31].

### Serology

An infectious bronchitis virus antibody test kit (IDEXX Laboratories, Westbrook, ME, USA) was used to analyse serum anti-IBV antibodies. The test was performed according to the manufacturer’s instructions. S/P ratios above 0.20 indicated positive results.

### Sample preparation

Cloacal swabs, kidney, bursa, and tracheal samples were prepared and processed as previously described [25]. Proventriculus and gizzard samples were washed in sterile phosphate-buffered saline (PBS) three times to remove feed residue and toxins [31]. The washed samples were then subjected to grinding with liquid nitrogen and diluted at a 1:5 w/v ratio in sterile PBS. The homogenates were frozen at −70 ℃.

### Histopathology and immunohistochemistry

After fixation for 48 h, tissue samples were embedded in paraffin wax, cut into 5 μm sections, stained with haematoxylin and eosin (H&E), and subjected to histopathological analysis by light microscopy. IBV in situ antigen detection was performed using immunohistochemical (IHC) staining and a monoclonal antibody targeting the N protein.

### Statistical analysis

Data were analysed using GraphPad Prism version 5.0 (GraphPad, La Jolla, CA, USA). The body weights and relative organ weights were analysed using the Kruskal‒Wallis test for nonparametric data, followed by Dunn’s multiple comparison test. The viral loads were analysed by two-way ANOVA, followed by Tukey’s multiple comparison test. A *P* value below 0.05 was considered significant, and a *P* value below 0.01 was considered highly significant.

## Results

### Isolation and phylogenetic analysis

**Figure 1:**
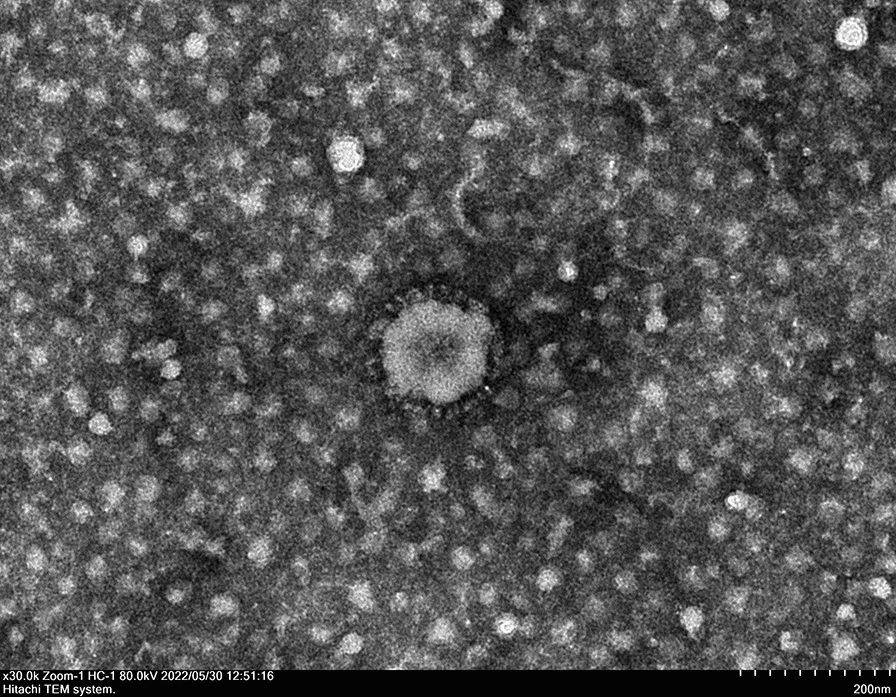
**Electron microscopic examination of CK/CH/GX/202109 virus particles, bar = 200 nm.**

**Figure 2:**
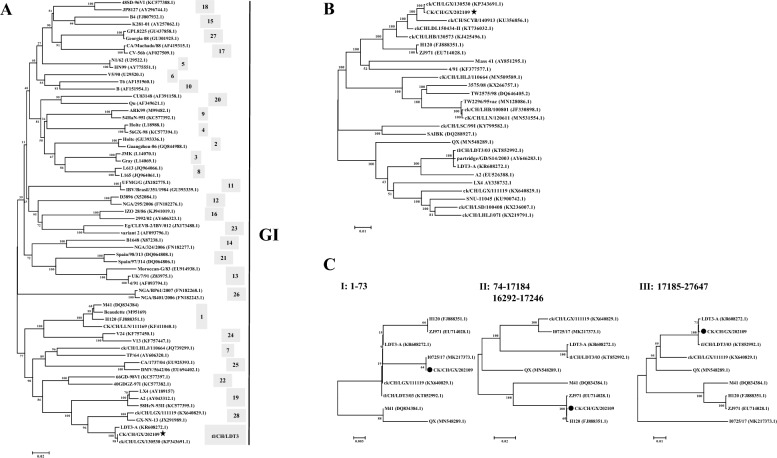
**Phylogenetic analyses of the *S1* gene (A), the whole genome (B) of the isolate CK/CH/GX/202109 (★) and other reference strains using the nearest neighbour-joining method with 1000 bootstrap calculations.** Phylogenetic trees of different genome regions (**C**) among CK/CH/GX/202109 (●), H120, tl/CH/LDT3/03 and other reference strains.

The CK/CH/GX/202109 strain (NCBI accession number OM970248) was isolated from the proventriculus and gizzard samples of three yellow broilers. Uncompromised IBV viral envelope and characteristic spike proteins were observed by negative staining electron microscopy (Figure 1). The phylogenetic analysis results based on the *S1* gene showed that this strain was clustered with the tl/CH/LDT3/03-like strain (99.6% similarity) (Figure 2A) and contained the same *S1* gene sequence as ck/CH/LGX/130530. Whole genome comparisons revealed that this strain was more closely related to the Massachusetts type strain (95.2% similarity to strain H120) than to the tl/CH/LDT3/03-like strain (92%) (Figure 2B).

### Recombination analysis

We determined the most likely recombinant fragments (*p* ≤ 10^–12^), the possible parental virus, and the beginning and ending points of those fragments. Recombination Detection Program version 4 (RDP4) and Simplot analyses showed that the CK/CH/GX/202109 isolate was closely related to the H120 (nucleotide position 74–17184) and ZJ971 (nucleotide position 16292–17246) strains based on fragment II of the *1ab* gene, and phylogenetic analysis confirmed the relationships between these IBV strains (Table 1, Figure 2C). The analyses showed that the isolate CH/CH/GX/202109 emerged from recombination events between the H120 strain, ck/CH/LGX/111119, ZJ971 and the tl/CH/LDT3/03-like strains. The 202109 strain shares 21 mutations with ck/CH/LGX/130530, including 9 synonymous mutations and 12 missense mutations (Table 2).

**Table 1:** **Recombinant breakpoints, genes, major and minor sequences of genomic recombination events of CK/CH/GX/202109.**

Breakpoints		Genes	Major sequence	Minor sequence	Detection methods (*p* value)
Beginning	Ending				
74	17184	*1ab*	ck/CH/LGX/111119 (KX640829.1)	H120 (FJ888351.1)	1.581 × 10^–321^ (RDP), 2.680 × 10^–266^ (GENECONV), 1.963 × 10^–299^ (BootScan), 7.441 × 10^–85^ (MaxChi), 1.109 × 10^–74^ (Chimaera), 7.824 × 10^–128^ (SiScan), 2.220 × 10^–15^ (3Seq)
16292	17246	*1ab*	tl/CH/LDT3/03 (KT852992.1)	ZJ971 (EU714028.1)	1.809 × 10^–96^ (RDP), 1.946 × 10^–94^ (GENECONV), 2.897 × 10^–89^ (BootScan), 4.663 × 10^–20^ (MaxChi), 4.580 × 10^–20^ (Chimaera), 5.574 × 10^–23^ (SiScan), 1.298 × 10^–12^ (3Seq)

**Table 2:** **Pairwise comparisons of nucleotide sequences of the whole genome of IBV isolate CK/CH/GX/202109 with the ck/CH/LGX/130530 strain.**

Strains	Genome position (1–27794 nt)																	
	*1ab*										*S2*	*3a*	*E*		*5a*	3'-UTR		
	*2251*^a^	*2857*	5028^b^	6384	*7153*	*8138*	8916	*13 721*	13 755	*18 791*	23 268	*23 848*	*24 315*	24 186	*25 667*	*27 374*	*27 376*	*27 448*
CK/CH/GX/202109	T	T	T	T	T	C	T	T	A	T	T	G	T	A	A	T	T	A
ck/CH/LGX/130530	C	C	C	C	C	A	C	C	G	C	C	T	G	G	C	C	G	G

^a^The missense mutations are shown in italics.

^b^Synonymous mutations are shown in normal font.

### Clinical signs

**Figure 3:**
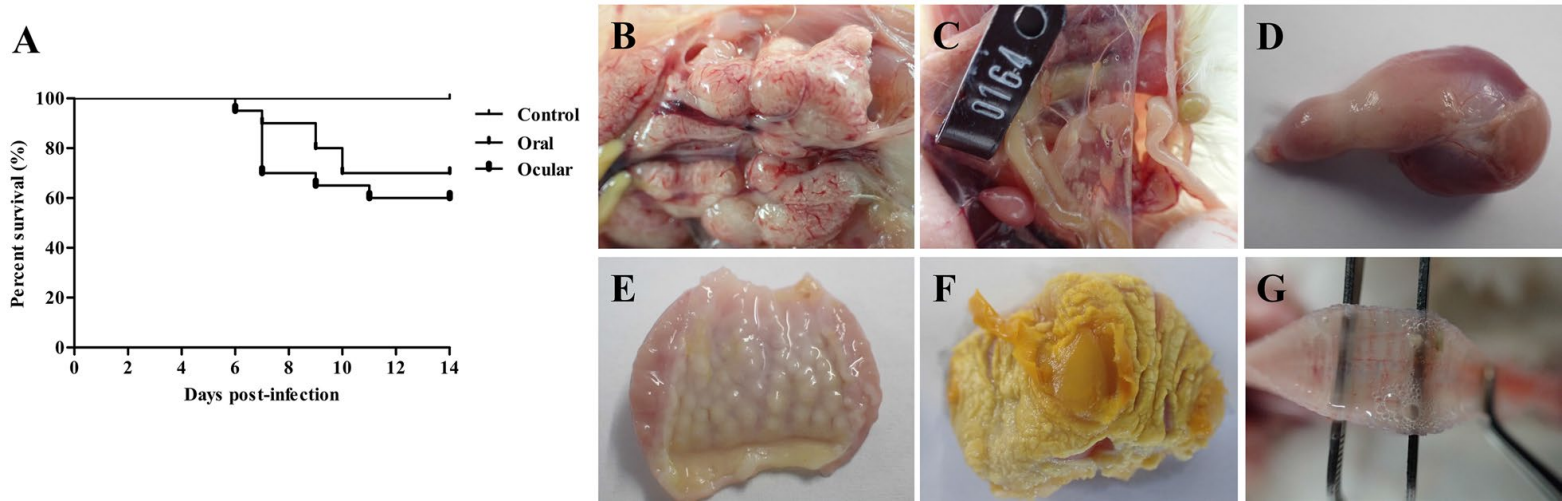
**The percentage survival of chicks in the 1-day-old groups and gross lesions in the 1-day-old and 28-day-old infected groups.** **A** Survival percentage of 1-day-old SPF chicks inoculated with the CK/CH/GX/202109 strain. **B** Nephritis in 1-day-old infected groups (7 dpi). **C** Air-sac lesions in the 1-day-old infected groups (7 dpi). **D** The proventriculus had a dilated gastric isthmus in the 1-day-old infected groups (14 dpi). **E** Unconspicuous papilla of the proventriculus and thickening of the wall in the 1-day-old infected groups (14 dpi). **F** Exfoliation of most of the endothelial corneum in the 1-day-old infected groups (7 dpi). **G** The tracheal membrane haemorrhage and small amount of mucus in 28-day-old infected groups (7 dpi).

Chicks in the 1-day-old infected groups showed clinical signs as early as 4 dpi, including depression, decreased feed intake, diarrhoea, huddling, ruffled feathers, and variable mortality, before beginning to recover at 12 dpi. The incidence of diarrhoea was highest at 5 dpi, with a maximum of nine chicks in the oral group and six in the ocular group affected. Mortality was observed at 6–7 dpi and 9–11 dpi in the infected groups, with a 30% rate in the oral group and a 40% rate in the ocular group (Figure 3A).

None of the chickens in the 28-day-old infected groups had any obvious clinical signs of infection. The control groups remained healthy throughout the study.

### Gross lesions

Most chicks in the 1-day-old infected groups exhibited diarrhoea before their death. The necropsy results showed that the primary clinical manifestation of IBV infection in the dead or euthanized chicks was nephritis (Figure 3B), in which urate deposits and swelling of the kidneys were observed; meanwhile, infected birds also exhibited air sacculitis (Figure 3C). Inflammation of the gizzard and atrophy of the bursa were also observed at both 7 and 14 dpi. The proventriculus was enlarged and had a dilated gastric isthmus (Figure 3D) and thickening of the proventriculus wall was also found at all observation times (Figure 3E). The papilla became inconspicuous or disappeared, and exfoliation of most of the corneum was also observed (Figure 3F). No obvious lesions were found in the gut tissues. The control group had no obvious lesions.

Haemorrhage and a small amount of mucus on the surface of the trachea were observed in the 28-day-old infected groups (Figure 3G). No other tissues were involved. No significant abnormalities were observed in the control group.

### Serology

With the exception of one bird in the ocular group, which was seropositive at 14 dpi, chicks in the 1-day-old groups were all negative for IBV antibodies. All birds in the 28-day-old infected groups were seronegative at 3 and 5 dpi. The IBV antibody positivity rate began to decline at 21 dpi and 14 dpi in the oral and ocular groups, respectively. Seroconversion did not occur in the control groups (Table 3).

**Table 3:** **Results of the serology.**

Experiments	Groups	Days post-infection						
		3	5	7	10	14	21	28
1-day-old	Control	0/20^a^	0/20	0/20	0/10	0/10	–	–
	Oral	0/20	0/20	0/18	0/6	0/6	–	–
	Ocular	0/20	0/20	0/14	0/9	1/8	–	–
28-day-old	Control	0/20	0/20	0/20	0/15	0/15	0/10	0/5
	Oral	0/20	0/20	2/20	11/15	12/15	7/10	3/5
	Ocular	0/20	0/20	3/20	14/15	12/15	6/10	2/5

^a^Numbers of chickens that seroconverted/the number of chickens tested.

### Body weight gain and relative organ weights

Body weight and weight gain following oral or ocular inoculation with IBV in the 1-day-old groups decreased significantly at 7 and 14 dpi (*P* < 0.05); however, pairwise comparisons performed at 14 dpi between the ocular and control groups that showed no significant difference. Compared to the control group, the increase in proventriculus and gizzard relative weight at 14 dpi was significantly higher in the infected groups (*P* < 0.05). A similar trend was observed in gizzard relative weight at 7 dpi in the oral group. The proventriculus weight at 7 dpi in the infected groups was significantly lower than that of the control group (*P* < 0.05). Compared to the control group, the bursa weight and bursa relative weight in the oral group decreased significantly at 7 and 14 dpi (*P* < 0.05) (Table 4).

**Table 4:** **Body weight, body weight gain, and relative organ weights (percentage of body weight) in the 1-day-old groups at 7 and 14 dpi.**

Dpi	Groups	Body weight (g)	Body weight gain (g)	Proventriculus weight (g)	Proventriculus relative weight	Gizzard weight (g)	Gizzard relative weight	Bursa weight (g)	Bursa relative weight
7	Control	78 ± 3^a^	37 ± 3^a^	0.87 ± 0.07^a^	1.12 ± 0.10	3.40 ± 0.35	4.39 ± 0.49^a^	0.27 ± 0.04^a^	0.35 ± 0.05^a^
	Oral	50 ± 9^b^	8 ± 9^b^	0.59 ± 0.09^b^	1.21 ± 0.14	2.97 ± 0.39	6.16 ± 1.35^b^	0.09 ± 0.04^b^	0.17 ± 0.07^b^
	Ocular	52 ± 10^b^	12 ± 11^b^	0.63 ± 0.11^b^	1.22 ± 0.18	3.12 ± 0.54	6.09 ± 1.30^ab^	0.13 ± 0.01^ab^	0.25 ± 0.04^ab^
14	Control	135 ± 10^a^	55 ± 13^a^	1.04 ± 0.09	0.77 ± 0.08^a^	4.13 ± 0.29	3.06 ± 0.19^a^	0.68 ± 0.16^a^	0.50 ± 0.11^a^
	Oral	84 ± 19^b^	23 ± 11^b^	0.92 ± 0.13	1.12 ± 0.18^b^	4.68 ± 0.72	5.95 ± 2.06^b^	0.28 ± 0.12^b^	0.32 ± 0.08^b^
	Ocular	101 ± 16^b^	41 ± 23^ab^	0.98 ± 0.16	0.97 ± 0.14^b^	3.79 ± 0.51	3.77 ± 0.32^b^	0.42 ± 0.14^ab^	0.40 ± 0.10^ab^

Means within a column and a time point with different lowercase superscripts were significantly different (*P* < 0.05).

Means were calculated based on live birds in each group.

Chickens in the 28-day-old oral administration group exhibited significant body weight and body weight gain decreases compared to age-matched control chickens at 7 dpi (*P* < 0.05). The gizzard relative weight at 14 dpi in the ocular group was significantly lower than that of the control group (*P* < 0.05). There were no differences in any other measures between groups at any other time point (Table 5).

**Table 5:** **Body weight, body weight gain, and relative organ weights (percentage of body weight) in 28-day-old groups.**

Dpi	Groups	Body Weight (g)	Body weight gain (g)	Proventriculus weight (g)	Proventriculus relative weight	Gizzard weight (g)	Gizzard relative weight	Bursa weight (g)	Bursa relative weight
7	Control	346 ± 19^a^	104 ± 9^a^	2.23 ± 0.18	0.65 ± 0.06	9.74 ± 0.73	2.81 ± 0.13	1.72 ± 0.32	0.50 ± 0.10
	Oral	333 ± 14^b^	88 ± 8^b^	2.24 ± 0.13	0.67 ± 0.04	8.84 ± 0.97	2.66 ± 0.27	1.76 ± 0.37	0.53 ± 0.11
	Ocular	374 ± 36^ab^	100 ± 10^ab^	2.31 ± 0.13	0.62 ± 0.07	9.40 ± 0.77	2.52 ± 0.12	1.93 ± 0.70	0.51 ± 0.17
14	Control	502 ± 36	120 ± 19	2.62 ± 0.27	0.52 ± 0.06	10.11 ± 1.55	2.01 ± 0.20^a^	2.88 ± 0.85	0.58 ± 0.17
	Oral	487 ± 28	131 ± 17	2.44 ± 0.30	0.50 ± 0.07	9.22 ± 1.20	1.90 ± 0.26^ab^	3.26 ± 0.65	0.67 ± 0.14
	Ocular	506 ± 34	128 ± 13	2.40 ± 0.20	0.48 ± 0.05	8.40 ± 0.70	1.66 ± 0.10^b^	2.75 ± 0.57	0.55 ± 0.14
21	Control	643 ± 99	121 ± 30	2.93 ± 0.41	0.46 ± 0.05	9.94 ± 1.70	1.55 ± 0.18	2.60 ± 0.30	0.41 ± 0.04
	Oral	622 ± 70	133 ± 20	2.49 ± 0.12	0.40 ± 0.05	9.65 ± 1.20	1.55 ± 0.09	2.96 ± 0.45	0.48 ± 0.07
	Ocular	649 ± 46	132 ± 10	2.70 ± 0.13	0.42 ± 0.02	9.82 ± 0.55	1.52 ± 0.13	2.48 ± 0.25	0.38 ± 0.04
28	Control	813 ± 100	145 ± 36	3.14 ± 0.27	0.39 ± 0.03	11.18 ± 1.55	1.38 ± 0.18	4.21 ± 1.01	0.51 ± 0.07
	Oral	811 ± 70	140 ± 20	2.98 ± 0.12	0.37 ± 0.05	11.10 ± 1.20	1.38 ± 0.09	3.99 ± 0.45	0.49 ± 0.07
	Ocular	787 ± 62	137 ± 23	3.18 ± 0.49	0.40 ± 0.07	11.22 ± 2.17	1.42 ± 0.24	3.04 ± 1.06	0.39 ± 0.15

Means within a column and a time point with different lowercase superscripts were significantly different (*P* < 0.05).

Means were calculated based on live birds in each group.

### Viral load

**Figure 4:**
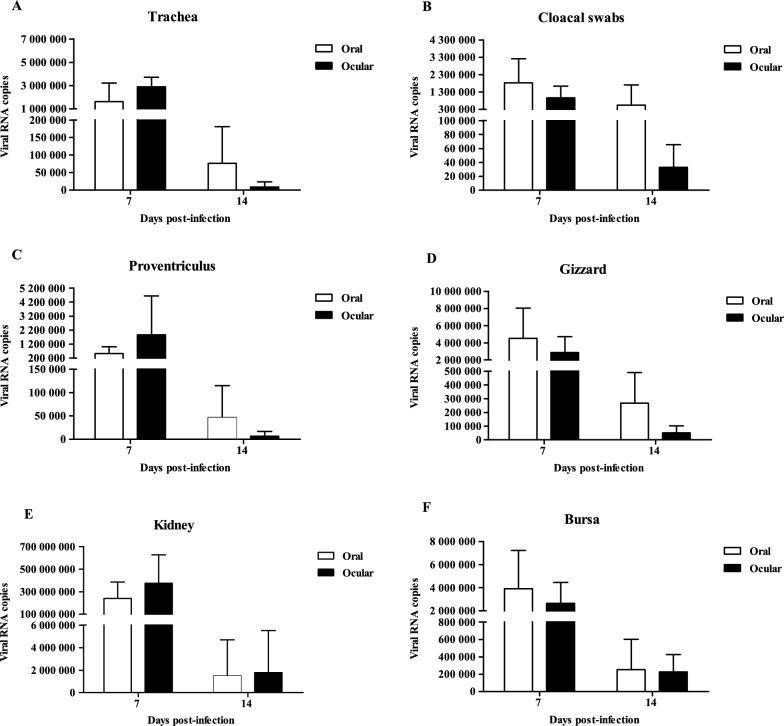
**Viral loads in different tissues from 1-day-old infected chicks.** The error bars indicate standard deviations.

No IBV was detected in any tissues harvested from the control groups at any time point. The mean viral genome copy numbers in the tissues of the 1-day-old infected groups were all above 10^6^ at 7 dpi, except for the cloacal swabs taken from the ocular group (above 10^5^) and those taken from the proventriculus from the oral group (above 10^5^); all copy numbers were higher than those at 14 dpi. The highest viral loads measured across all samples occurred in kidney tissues at 7 dpi (above 10^8^) (Figure 4).

**Figure 5:**
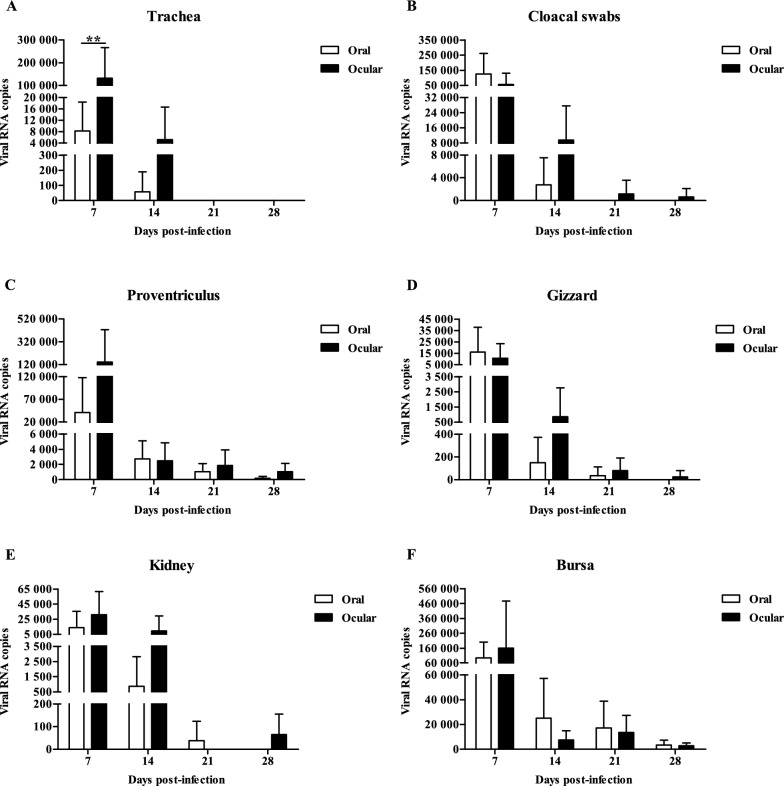
**Viral loads in different tissues from 28-day-old infected chickens.** The error bars indicate standard deviations. Data were analysed using two-way ANOVA, ** *P* < 0.01 indicated a highly significant difference.

The mean viral loads in the tracheal samples from the 28-day-old ocular inoculation group were significantly higher than those of the oral group at 7 dpi (*P* < 0.01). The mean viral loads in the tissues of the infected groups peaked at 7 dpi and decreased rapidly at 14 dpi. The maximum viral genome copy number in the trachea, proventriculus and bursa from the ocular group and cloacal swabs from the oral group were all above 10^5^ at 7 dpi (Figure 5).

### Histopathology

**Figure 6:**
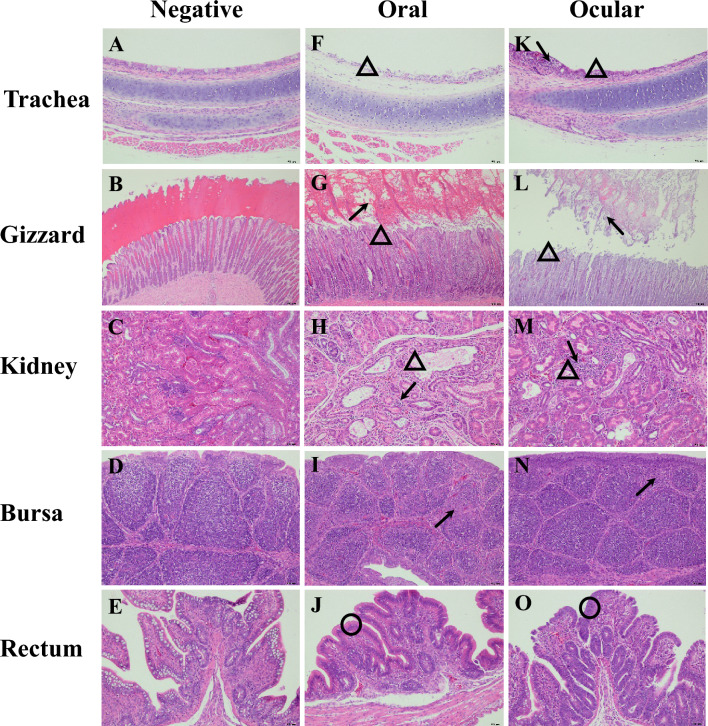
**Histopathologic changes detected in different tissues in the 1-day-old groups.** **A**–**E** tissues in the negative group (7 dpi). **F**–**J** tissues in the oral group. **K**–**O** tissues in the ocular group. **F** and **K** black arrows indicate inflammatory cellular infiltration in the trachea. The empty triangles indicate upper mucosa necrosis at different degrees (7 and 14 dpi). **G** and **L** black arrows indicate necrotic and exfoliated cells in the horn-like structure of the gizzard. The empty triangles indicate that the mucosal epithelium is moderately or seriously denatured and necrotic (7 dpi). **H** and **M** black arrows indicate inflammatory cellular infiltration in the kidney. The empty triangles indicate that the renal tubules were mildly to moderately denatured (7 and 14 dpi). **I** and **N** black arrows indicate a mild decrease in the number and volume of lymphocytes in the bursa (7 dpi). **J** and **O** The empty circles indicate mildly to moderately reduced goblet cells in the rectum (7 dpi).

No IBV-related lesions were observed in the control group (Figures 6A–E). The lesions were similar between the 1-day-old groups infected via the oral and ocular routes in terms of location and severity. The cells in the upper mucosa of the tracheas exhibited varying degrees of necrosis, and a large number of infiltrating inflammatory cells were also found in the tracheas of the ocular group (Figures 6F and K). The mucosal epithelia were moderately or severely denatured and necrotic in the gizzards of both infected groups, and necrotic and exfoliated cells were found in the hornlike structure of the gizzards (Figures 6G and L). Regarding the kidneys, mild to moderate degeneration of the uriniferous tubules, as well as inflammatory cell infiltration, was observed in both groups (Figures 6H and M). Bursal lesions were characterized by a slight decrease in the volume of the lymphoid follicles and the number of lymphocytes (Figures 6I and N). A clear reduction in goblet cells was characteristic of rectal tissue lesions (Figures 6J and O).

Lesions in the 28-day-old oral and ocular groups were mainly found at 7 dpi. Moderate cell degeneration in the upper mucosa and inflammatory cell infiltration were observed in the tracheal lesions. Varying degrees of infiltration of inflammatory cells were observed in the renal interstitium of chickens in both infected groups. In the bursa from the oral group, lesions were characterized by a slight decrease in the volume of the lymphoid follicles and the number of lymphocytes.

### Immunohistochemistry

**Figure 7:**
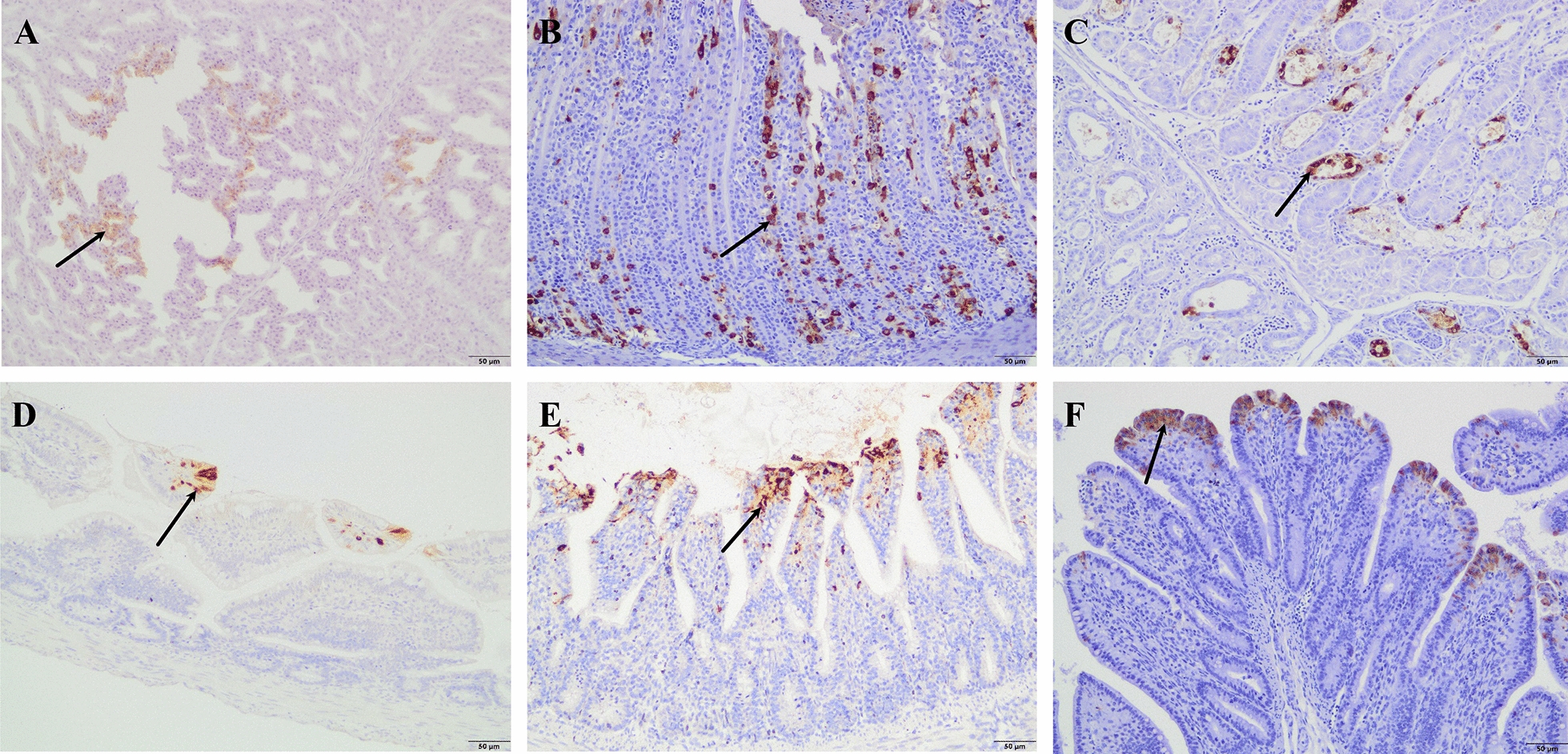
**IBV antigens were detected in tissues from infected groups by immunohistochemistry; black arrows indicate the viral antigens.** **A** The epithelial cells of proventriculus tubules in the 1-day-old oral group (14 dpi). **B** Mucosal epithelium of the gizzard in the 1-day-old infected groups (7 dpi). **C** Renal tubular epithelial cells of the 1-day-old oral group (14 dpi). **D** Epithelial cells in the jejunum of the 1-day-old and 28-day-old ocular groups (7 dpi). **E** Epithelial cells in the ileum of the 1-day-old and 28-day-old ocular groups (7 dpi). **F** Epithelium cells in the rectum of the 1-day-old infected groups (7 dpi) and 28-day-old ocular group (7 dpi).

No IBV antigens were observed in the control groups. Specific viral antigens were found in the proventriculus, gizzard, kidney and rectum of the 1-day-old orally infected group and in the gizzard, jejunum, ileum, and rectum of the ocular group, but no virus was detected in the trachea, bursa, duodenum, and caecum. The virus was detected in the jejunum, ileum, and rectum at 7 dpi in the 28-day-old ocular group (Figure 7).

## Discussion

Recombination has been found to shape the evolution of the tl/CH/LDT3/03 type strains [3, 21, 32]. The RDP4 results confirmed that the CK/CH/GX/202109 strain emerged through recombination events between the H120, ck/CH/LGX/111119, ZJ971 and tl/CH/LDT3/03 strains, as has been shown for other tl/CH/LDT3/03 type strains. Previously, when attenuated or inactivated IBV vaccines have been applied to chickens reared on large-scale farms with high bird densities and incomplete “all-in/all-out” management, recombination events between epidemic and vaccine strains have been found in GI-1 [33], GI-7 [2], GI-19 [34, 35] and other genotypes. These observations indicate that this phenomenon is becoming a trend, and close attention should be given to antigenic changes and vaccine selection. Furthermore, CK/CH/GX/202109 shares an *S1* gene sequence with ck/CH/LGX/130530, and it is essential for the initial attachment of the virus to a host cell. This sequence is applied for IBV strain genotyping [12]. Compared to the whole genome sequences, 21 point mutations were found in the *1ab*, *S2*, *3a*, *E*, *N*, and *5a* genes and the 3-UTR, which may result in this strain having higher pathogenicity compared to the strain ck/CH/LGX/130530 with low pathogenicity. Similar findings have been made for strain 3575/08, in which there were gene mutations that conferred stronger respiratory and renal pathogenicity than 2575/98 [36], suggesting that strain CK/CH/GX/202109 also evolved via the accumulation of point mutations.

IBV was first identified as one of the most important respiratory pathogens in chickens, and live virus can be isolated from multiple tissue types. In this study, viral copy numbers were all above 10^5^ in the trachea, proventriculus, gizzard, kidney, bursa, and cloaca swabs taken from the 1-day-old oral and ocular groups at 7 dpi. However, the specific ck/CH/LGX/130530 strain was not detected. Moreover, histopathological examination of the lesions and IHC results in infected chickens indicated that this strain was highly pathogenic and could replicate in the proventriculus, gizzard, and intestines, demonstrating its broad tissue tropism. Interestingly, the pathogenicity of this strain by oral or ocular inoculation was similar. The striking difference was in the replication in the intestines, which may have been due to the infection route. Findings on the pathogenicity of other variants were consistent [4, 26, 27] in demonstrating a tendency for the IBV strain to infect other tissues that had not been considered, thus reminding us to be prepared for unusual IBV tissue tropisms in which recombination and mutations of the microbial genome may be the first consideration. The binding of the virus to a cellular receptor is the first step in viral infection. *S2* gene sequence variability was reported to affect S1 subunit-specific antibody binding [37] and, in conjunction with S1, be responsible for cellular tropism [13, 38]. The interplay between the S1 and S2 spike subunits might determine the attachment and host range of IBV [39–41]. The furin cleavage site plays an important role in virus pathogenicity, and the addition of a furin-S2 cleavage site has been shown to alter virus cell tropism [42], indicating that the role of the S2 protein should be considered. Moreover, non-structural 1ab [9] and accessory proteins, including 3a, 3b [43], 5a [15], and 5b [16], may also be determinants of pathogenicity, suggesting that alterations in the tissue tropism or virulence of IBV may be affected by other proteins in addition to S1. Thus, determining the relationships between these genes in terms of pathogenicity requires further study.

Chickens of all ages are susceptible to IBV infection. In the present study, the CK/CH/GX/202109 virus at a dose of 10^6^ EID_50_ resulted in 30% and 40% mortality rates when administered via oral and ocular routes, respectively, in 1-day-old chicks, which was higher than that of strain ck/CH/LGX/130530 (0%) but lower than the 70% rate in strain tl/CH/LDT3/03 administered at a dose of 10^5^ EID_50_ [3]. However, no mortality was recorded in 28-day-old chickens, indicating that this strain is more virulent in young chicks. Additionally, almost no seroconversion was observed in the 1-day-old infected groups until 14 dpi. In contrast, seroconversion occurred at 8 dpi in chicks inoculated with ck/CH/LGX/130530 or tl/CH/LDT3/03 [3]. A previous study demonstrated that bursectomized chickens experienced more severe and longer illnesses than intact chickens and that IgG was first detected at 9 dpi [44]. The viral load in the bursa in the 1-day-old infected groups was above 10^6^ at 7 dpi and above 10^5^ at 14 dpi by qRT‒PCR, which was higher than that seen in infections with the I0305/19 strain with multiorgan tropism in embryonated eggs [26]. In addition, the number of lymphocytes in the bursa and the bursa relative weight decreased, but no special viral antigens were found in the bursa because of mistimed sampling and little live virus. It is presumed that functional changes in the bursa of chicks infected with strain 202,109 are mostly similar to those of bursectomized chickens, which do not produce specific antibodies against IBV. Thus, more immunological research is needed on the immunosuppression of IBV. Conversely, the viral load of the bursa was between 10^4^ and 10^5^ at 7 dpi and below 10^4^ at 14 dpi in the 28-day-old infected groups, and chickens began to seroconvert by 7 dpi. No significant difference was observed in the relative bursa weights when comparing the treatment and control groups, and it was suggested that a certain level of viral load may be indicative of lesions in the bursa. Interestingly, endothelium corneum and gastric mucosa exfoliation in 1-day-old infected groups were observed for the first time. The IHC results confirmed the presence of IBV antigens in the gizzard mucosa.

Usually, enteric tissue infection does not manifest clinically [45, 46]. In this study, reductions in the number of goblet cells in the rectum were obvious. For isolate G, high viral titres were found in all enteric tissues except for the jejunum, and desquamation of epithelial cells from the villus tips was observed [47]. The experimentally infected chickens exhibited reduced body weight and diarrhoea. IHC staining of IBV samples provided evidence supporting the digestive and enteric tropism of this strain. It is important to distinguish IBV infection from runting-stunting syndrome, which is seen clinically as a consequence of adenovirus or reovirus infection [48]. In contrast, neither body weight changes nor diarrhoea was observed in the 28-day-old infected groups; these groups also did not show gross pathological lesions in the digestive and enteric tissues. Only special viral antigens were detected in the jejunum, ileum, and rectum at 7 dpi in the ocular group, indicating that 28-day-old chickens were more resistant to infection with this strain than 1-day-old chicks. However, it is noteworthy that lymphocyte infiltration was observed in the trachea and kidney, and IBV antigens were not detected in the trachea, perhaps due to exfoliation of the mucosal epithelium. Thus, slight damage to the trachea may occur in the absence of visible symptoms, which increases the susceptibility to bacteria, other viruses, and mycoplasma [49–51].

This study identified an IBV variant, CK/CH/GX/202109, that clustered with the tl/CH/LDT3/03-like strains and emerged from recombination and mutations. The CK/CH/GX/202109 strain was lethal in 1-day-old SPF chicks after oral and ocular administration, with 30% and 40% mortality rates, respectively. Trachea, proventriculus, gizzard, kidney, bursa, and cloacal swabs were positive for viral RNA at 7 dpi in both the 1-day-old and 28-day-old infected birds. Multiorgan tropism was found in 1-day-old infected chicks, and virus antigens were detected in the ileum, jejunum, and rectum of the 28-day-old ocular group.

## Data Availability

All data generated during this study are included in the published article.
